# Cien años de vacunación en Yucatán, México, 1802-1902

**DOI:** 10.1590/S0104-59702026000100026

**Published:** 2026-08-07

**Authors:** Ricardo Manuel Wan Moguel

**Affiliations:** i Red de Historia Demográfica. Mérida – Yucatán – México. manuelwanmoguel@gmail.com

**Keywords:** Vacuna, Viruela, Virus, Epidemia, Yucatán (México), Siglo XIX, Vaccine, Smallpox, Virus, Epidemic, Yucatán (Mexico), Nineteenth century

## Abstract

Este artículo analiza la introducción, propagación y desafíos de la vacuna en Yucatán, México, desde su introducción en 1802 hasta 1902, cuando se realizaban campañas recurrentes de vacunación. A partir de fuentes cuantitativas y cualitativas abordamos la llegada de la vacuna y los primeros esfuerzos de las autoridades para propagarla por la región. Estudiamos la resistencia de la población a vacunarse y las estrategias del Estado para difundir el antídoto. Presentamos gráficas del número de las personas inoculadas, principalmente a finales del siglo XIX. Argumentamos que, a pesar de los esfuerzos iniciales, múltiples contratiempos impidieron el desarrollo eficiente de las campañas, pero poco a poco la vacunación se consolidó.

## Abstract (en)

This article analyzes the introduction, spread, and challenges of vaccination in Yucatán, Mexico, from its introduction in 1802 through 1902, a period marked by recurrent vaccination campaigns. Using quantitative and qualitative sources, I approach the arrival of the vaccine and the initial efforts made by authorities to promote it throughout the region. I studied the population’s resistance to vaccination and the state’s strategies to disseminate the antidote. I also present graphs showing the number of people inoculated, mainly at the end of the nineteenth century. I argue that despite the initial efforts, multiple setbacks hindered the effective development of the vaccination campaigns, but the vaccination became consolidated over time.

En la actualidad la península de Yucatán está conformada por los estados de Campeche, Quintana Roo y Yucatán. Sin embargo, durante la Colonia, ellos formaban una sola provincia (Rodríguez Losa, 1989, p.21). Tras la Independencia, la península se incorporó al Imperio Mexicano, encabezado por Agustín de Iturbide. Con el paso del tiempo, y a pesar de las desavenencias entre las autoridades locales y las nacionales, la región permaneció incorporada al Estado mexicano como una sola entidad hasta mediados del siglo XIX. En 1857, Campeche se independizó de Yucatán, naciendo así dos entidades federativas (Rodríguez Losa, 1989, p.107). Por otra parte, el 5 de noviembre de 1901, Manuel González, Cosío, secretario de Gobernación durante el gobierno de Porfirio Díaz, presentó ante la Cámara de Diputados del Congreso de la Unión una iniciativa de reforma constitucional con el propósito de erigir el territorio federal de Quintana Roo en la porción oriental de la península de Yucatán. Dicha propuesta culminó el 24 de noviembre de 1902, cuando Porfirio Díaz decretó su creación. Con ello, quedaron conformadas las tres entidades que, hasta la actualidad, integran la península de Yucatán (Rodríguez Losa, 1991, p.41).

El presente artículo tiene como objetivo analizar la propagación de la vacuna en Yucatán durante sus primeros cien años de historia, abarcando el periodo comprendido entre 1802, año en que fue introducida a la región a través de la expedición filantrópica comandada por Francisco Xavier Balmis, y 1902, cuando las instituciones del estado yucateco ya se habían consolidado y llevaban a cabo campañas de vacunación de manera constante. Aunque el tema de la vacunación ha sido estudiado por diversos autores en Latinoamérica, México y Yucatán (Agostoni, 2016, Alcalá, 2008, 2022; Caffarena, 2016; Carrillo, 2002, 2010; Di liscia, 2021; Fernandes, 2003), este trabajo busca ofrecer un panorama integral sobre un siglo de prácticas vacunales en Yucatán, México. Se estudia bajo una perspectiva institucional y social, haciendo énfasis en el papel que desempeñaron las autoridades locales en su difusión, así como la resistencia de la población a recibir la vacuna. Esta investigación se enfoca en la región peninsular, particularmente en Yucatán, pues la información recabada proviene de este estado. Se consultó el Archivo General del Estado de Yucatán y la Biblioteca Yucatanense, donde se obtuvieron documentos relacionados con las campañas de vacunación y estadísticas vitales para comprender la propagación de la vacuna.

El texto se divide en cinco apartados: primero se da un panorama de la administración de la salud en el periodo en cuestión para comprender las instituciones que estuvieron a cargo de la vacunación. Seguidamente, se analiza la etiología de la viruela y el conocimiento médico acerca de esta enfermedad. En la tercera sección se estudian los remedios recomendados para detener la epidemia, la implementación de la variolización y la llegada de la vacuna a Yucatán, con la campaña del médico valenciano Francisco Xavier Balmis. En el cuarto apartado se estima, a partir de los datos estadísticos, el número de personas vacunadas desde finales del siglo XIX y principios del siglo XX. Finalmente, se destacan los hallazgos del trabajo.

## La administración de la salud

Desde la época colonial, las instituciones de caridad, estaban bajo la administración de la Iglesia, que brindaba asistencia a los enfermos y desfavorecidos. Sin embargo, con la institucionalización de las intendencias en 1786, se inició un proceso de secularización de la beneficencia, consolidado a mediados del siglo XIX. La promulgación de la Constitución de Cádiz en 1812 estableció un marco jurídico que facultaba a los ayuntamientos para asumir la responsabilidad en este ámbito (Castillo, 2002). A partir de entonces, los ayuntamientos se encargaron de las cuestiones de salud e higiene en las ciudades, además de velar por la propagación y suministro de la vacuna. Para atender los problemas sanitarios de la región, se crearon juntas de sanidad, responsables de gestionar las enfermedades prevalentes en la península de Yucatán durante el siglo XIX. La Junta General de Sanidad, presidida por el gobernador, operaba a través de dos comisiones permanentes ubicadas en Campeche y Mérida, encargadas de regular las actividades de salubridad en sus respectivas jurisdicciones (Peniche Moreno, 2016, p.57-138).

Aunque estas juntas dependían del gobernador, los ayuntamientos desempeñaban un papel fundamental en la regulación de sus actividades. Las ordenanzas municipales de Mérida (capital de Yucatán) de 1861 estipulaban que los ayuntamientos debían gestionar las epidemias, vigilar la higiene pública, implementar medidas profilácticas frente a enfermedades endémicas, establecer cuarentenas y convocar reuniones de emergencia (Wan Moguel, 2024, p.348-359). Durante el gobierno de Porfirio Díaz (1876-1911), el gobierno estatal se fortaleció y asumió un papel más activo en materia de salubridad. En Yucatán, la estructuración del sistema sanitario alcanzó un punto culminante en enero de 1894, con la fundación del Consejo de Salubridad, consolidando así una organización clara y eficiente del sector salud. Esta institución tenía funciones muy precisas en materia de salud: combatía las epidemias, supervisaba comercios expendedores de leche, carne y productos alimenticios, inspeccionaba las farmacias, examinaba las condiciones higiénicas de casas y negocios, regulaba las industrias clasificadas como peligrosas y organizaba campañas de vacunación. No obstante, la propagación de la vacuna también enfrentó importantes limitaciones presupuestarias, ya que desde 1847 se inició un prolongado conflicto entre las autoridades estatales y la población maya, conocida como la Guerra Social Maya, o Guerra de Castas, el cual se extendió hasta 1902 y afectó toda la península, principalmente la región sur y oriente. Durante este periodo, los recursos económicos fueron prioritariamente destinados a acciones militares para sofocar la sublevación, lo que ocasionó un descuido parcial de diversas iniciativas de salud pública, entre ellas las campañas de vacunación (Wan Moguel, 2024, p.33).

## Viruela y epidemias

La viruela es una enfermedad altamente infecciosa y contagiosa, causada por el virus *Variola*. Se transmite de persona a persona, por medio de la inhalación de las pequeñas gotas emanadas de las vías respiratorias de los enfermos que contienen el virus desprendido de lesiones de la mucosa bucofaríngea. El periodo de incubación va entre los siete y los 17 días y pasa por cuatro periodos: incubación, prodrómico o sintomático inicial, eruptivo o de contagio y resolución o de muerte. En cuanto a su origen, hay indicios de que estuvo presente en el antiguo Egipto según los estudios que se hicieron de las lesiones de la momia de Ramsés V en el 1160 a.C. Asimismo, se ha detectado su presencia en la dinastía Zhōu de China desde 1122 a.C y en Europa desde el siglo V (Valdés Aguilar, 2010, p.27-30).

En los territorios americanos, se data su origen en 1529, cuando Pánfilo de Narváez introdujo personas de origen africano con el *Variola virus* (Thompson, 1997, p.78). En la península de Yucatán, hay investigaciones que vislumbran que en la Colonia la viruela ya tenía un nombre en el idioma maya, que permitía identificar el mal entre las personas. Se trata del término *habal*, que significaba literalmente “el que tiene viruela”. Según algunos autores como Eric Thompson, su presencia se remonta al siglo XVI, entre 1500 y 1520 (Wan Moguel, 2023, p.34). Otra palabra maya usada para nombrarla es *k’aak*, “fuego” (Birman Furman, 1996, p.12).

Durante el siglo XIX, la forma en que se entendía la viruela en Yucatán muestra cómo el conocimiento médico y el miedo social estaban profundamente relacionados. En publicaciones como *La Emulación*, de 1874, se describía la enfermedad con precisión médica, mencionando síntomas como fiebre, vómito, dolores lumbares y la aparición de pústulas por todo el cuerpo, incluso en el rostro. Sin embargo, más allá del diagnóstico clínico, estas descripciones también reflejan el temor que causaba una enfermedad tan visible y estigmatizante. La posibilidad de quedar marcado de por vida generaba angustia, especialmente entre quienes sobrevivían. Los tratamientos recomendados, que incluían bebidas aromáticas, ungüentos y emplastos con mercurio, muestran cómo la medicina de la época intentaba aliviar no solo los síntomas físicos, sino también la preocupación social frente a una enfermedad que dejaba huellas duraderas. En ese sentido, la viruela era vista no solo como un problema de salud, sino también como una amenaza que impactaba el cuerpo, la imagen personal y la vida cotidiana (Wan Moguel, 2024, p.183-196).

En la península de Yucatán hubo varias epidemias a lo largo de la historia. Durante la Colonia se han identificado brotes en el siglo XVI, como los ocurridos en 1573 y 1575. En el siglo XVII se presentó en 1609, 1627, 1631, 1648, 1656 y 1659 (Alcalá, 2010, p.76). En las postrimerías del siglo XVIII se ha analizado el impacto demográfico de la epidemia desatada en 1782 (Falla, 2013, p.334), y en 1793 se dio un brote generalizado en diversas regiones del virreinato de la Nueva España, y para el caso de la península, se propagó rápido desde Mérida hasta Campeche, por el camino real que unía ambas ciudades (Sánchez Moo, 2022, p.8). En el siglo XIX hubo epidemias en 1825, 1833, 1842, 1846, 1852, 1855, 1857, 1864 y 1874-1875 (Wan Moguel, 2023, p.81-82). De todos ellos, solamente se ha analizado el impacto demográfico de la epidemia de 1874-1875, que causó la muerte de aproximadamente 15 mil personas solamente en el estado de Yucatán. En los periodos de 1900-1901, la viruela provocó más de setecientos defunciones en el partido de Mérida, que fungía también como capital de Yucatán (Wan Moguel, 2024, p.183-227). No obstante, aún es necesario profundizar en el estudio de su impacto en el resto del estado, así como determinar una fecha precisa en la que se pueda establecer que la viruela dejó de representar una amenaza letal para la población.

## La introducción de la vacuna

La variolización o inoculación, procedimiento que consistía en introducir a las personas sanas el *Variola virus* a través de costras sanas o inserción de estas, fue quizá iniciado en China y la India hace alrededor de dos mil años. En 1721 fue llevado a Europa por Lady Wortley-Montague, esposa del embajador inglés establecido en Turquía. Su práctica fue recurrente y fue llevado a las colonias de los ingleses, portugueses y españoles, incluido a la Nueva España (Valdés Aguilar, 2010, p.33). Cabe señalar que además de la variolización, se tiene conocimiento de diversos “remedios” a los que recurría la población autóctona para curar los malestares provocados por la enfermedad. Se recomendaba “sangrar” a las personas, y darles diferentes sustancias y plantas medicinales locales. Para las llagas, se usaba la ruda cocida en vino que se debía untar (Wan Moguel, 2023, p.35). Sin embargo, las cosas comenzaron a cambiar en las postrimerías del siglo XVIII, cuando el médico Edward Jenner hizo los primeros experimentos con material infectado de las lesiones de *cowpox* al niño James Phipps, de 8 años. Sus investigaciones se publicaron en 1798, generando discusiones científicas y académicas, pero sus ideas de la vacunación poco a poco ganaron adeptos entre la población pues se publicaron folletos, cartillas y material que buscaron convencer a la gente para que se aplicaran la vacuna (Barona, 2004, p.23-26).

La Nueva España no quedó exenta del descubrimiento, aunque la vacuna se introdujo con la Real Expedición Filantrópica de Francisco Xavier Balmis, mandada por el rey Carlos IV. Para ello, se usaron varios facultativos, empleados y 22 niños que fueron los reservorios del virus para ser transmitido posteriormente, de brazo en brazo. Susana Ramírez señala que hubo varias propuestas de rutas y, a pesar de que se aprobó una el 26 de mayo de 1803, ninguna fue seguida por Balmis, pues la urgencia de propagar la vacuna determinó la modificación de esta. Además, resulta importante mencionar que la expedición se debe de estudiar en tres momentos, pues en la capitanía de Venezuela se divide en dos. Por ello, lo conveniente es analizar los diferentes tramos: la expedición conjunta, la de Balmis y la de Salvany. Así, la expedición comenzó en Madrid el 7 de septiembre de 1803, trasladándose luego a la Coruña, donde se preparó la travesía (Ramírez Martín, 2004, p.37-38), que siguió por Canarias, Puerto Rico y Caracas. De esta provincia salieron por el puerto de Guaira en dos direcciones. A la América Meridional, a cargo del subdirector Salvany, y hacia la Habana y Yucatán, con Balmis a la Cabeza. En este último punto, la expedición se volvió a dividir. Francisco Pastor, uno de los acompañantes de Balmis, partió del puerto de Sisal hacia Villahermosa y de ahí a Chiapas, Oaxaca y Guatemala. Los otros fueron hacia Veracruz, y el resto del virreinato de la Nueva España.

A la península de Yucatán, la vacuna llegó antes que la expedición en la ciudad de Campeche, aunque no se introdujo al resto de la Intendencia (Morales Reyes, 2022, p.34-35, Sánchez Moo, 2024, p.75-77). Entre 1800 y 1810, Benito Pérez Valdelomar ocupaba el puesto de capitán general e intendente de Yucatán, quien informó a los facultativos la llegada de Balmis a la entidad a través de Cuba. La expedición llegó a Sisal el 25 de junio de 1804 y cuatro días más tarde a la ciudad de Mérida (Ramírez Martín, 2004, p.40), donde Valdelomar ordenó que la expedición recibiera todas atenciones necesarias en muestra de la gratitud y la labor de la campaña. De hecho, fueron alojados y se les proveyó de camas, manutención y asistencia. Los gastos generados corrieron a cargo de la Real Hacienda. Valdelomar facilitó también a cuatro jóvenes para extender la vacunación a Guatemala. Los galenos de la entidad recibieron instrucciones de cómo se debía de vacunar, pues hay que recordar que la Expedición no solamente tenía el objetivo de propagar la vacuna, sino de crear juntas locales que sirvieran para conservar y difundir el preservativo. Tras ello, Alejo Dancourt, un médico de origen francés y radicado en Mérida, fue nombrado como encargado en la capital y en la subdelegación para preservar y propagar la vacuna.

Tiempo después, en 1806, José Serna se encargó de propagar la vacuna en los pueblos de Sotuta, Tizimín (centro-sur y noreste del estado) y Tihosuco (actualmente perteneciente al estado de Quintana Roo), cuya labor era remunerada con seis pesos diarios. Tres años después, Dancourt, según su propio testimonio, vacunó a más de tres mil niños en Valladolid (oriente de Yucatán), pero no hemos encontrado documentos relacionados con esa inoculación masiva. Al retirarse, dejó encargado a Antonio Belchd para continuar con la labor, con una remuneración de treinta pesos mensuales, que corrió a cargo del municipio. La vacunación se extendió a otros pueblos, como Ticul al sur de Yucatán (Wan Moguel, 2023, p.83-86).

A principios de 1811, se establecieron juntas especiales destinadas a la propagación del fluido, lo que resultó fundamental para garantizar la conservación y disponibilidad de la vacuna. Como parte de esta iniciativa, se designó a José Taxo como secretario. Sin embargo, la salida del gobierno de Valdelomar ese mismo año, sumada a la renuncia del secretario, disminuyeron los esfuerzos para propagar la vacuna, aunque todavía se debe de esclarecer lo sucedido en ese periodo.

En 1813, Sebastián Sotomayor fue nombrado como comisionado encargado de la distribución de la vacuna en Hecelchakán, una región perteneciente a Campeche. Posteriormente, en 1815, José González recorrió las subdelegaciones de la provincia con el objetivo de extender la cobertura de la vacunación, recibiendo como remuneración medio real por cada niño vacunado.^1^ Un año antes, Cipriano Blanco se encargó de propagar la vacuna en Campeche, quien dejó un testimonio de una persona que recibió la vacuna en esos años:

José Flores, mestizo del pueblo de Hecelchakán de edad de 4 años, fue vacunado con cuatro incisiones en cada brazo el día 6 del presente con el fluido de una vacuna perfecta: al tercer día de su inoculación se presentó en todo el tronco de su cuerpo una abundante erupción variolosa: los granos vacunos ingeridos sufrieron un atraso de cinco días más que lo ordinario, y no presentaron hasta el octavo día ningún signo de su verdadero carácter: en concepto de creerlos perdidos volvió a revacunarse el día 14 de dicho mes, y este mismo día. Después de operado se desenvolvieron los granos primeramente inoculados con toda su energía desapareciendo al propio tiempo la erupción indicada, y a los tres días inmediatos salieron con toda perfección los de la segunda vacunación, caminando dicho joven… (Estado…, 1814, p.3-4).

El testimonio anterior deja entrever lo ocurrido en el cuerpo, al recibir la vacuna y la necesidad de realizarse la revacunación ante cualquier sospecha de que el antídoto no surtiera el efecto esperado. Cabe recordar que, según el relato de Alejo Dancourt, desde el inicio de las campañas de vacunación, personas de diversos sectores de la población acudieron a recibir el antídoto contra la viruela. Durante este periodo inicial, las fuentes oficiales indican que la oposición a la vacuna fue mínima, en parte gracias a la intervención activa de los párrocos, quienes desempeñaron un papel clave al persuadir a aquellos que se mostraban reacios a la inoculación (Wan Moguel, 2023, p.83-86). Sin embargo, esta afirmación requiere ser comprobada mediante un análisis detallado de los registros de vacunación, con el fin de determinar la cantidad exacta de personas que efectivamente recibieron la inmunización. Esta necesidad se hace más evidente al considerar que en 1825 ocurrió una epidemia de viruela particularmente mortífera, lo que podría sugerir fallas en la eficacia de la vacuna o, más probablemente, una insuficiente propagación de esta entre la población. Por lo tanto, la revisión y sistematización de estos reportes resulta esencial para evaluar el verdadero alcance y éxito de las campañas de vacunación en esta etapa inicial.

Con el paso del tiempo, comenzaron a surgir informes que evidencian la resistencia de la población a someterse al proceso de vacunación. Un documento gubernamental de 1848 señala que muchos padres de familia se negaban a llevar a sus hijos a ser vacunados, principalmente por dos razones: el temor al procedimiento y la desconfianza hacia las personas encargadas de administrarlo. Este fenómeno no solo revela una falta de información generalizada respecto a los beneficios de la inmunización, sino también la insuficiencia de estrategias educativas para generar confianza en las comunidades (Rejón, 25 feb. 1848, p.1-2). La resistencia dejaba entrever la importancia de que el Estado interviniera para informar a la población sobre los beneficios de la vacunación, evitando así nuevos brotes epidémicos y asegurando que los infantes no fueran ocultados ni se les negara el acceso al antídoto.

En 1843 se publicó la *Cartilla o instrucción sobre la vacuna escrita por Miguel Muñoz, profesor cirujano y comisionado por la superioridad para la observación y propagación de este precioso antídoto*, que reimprimió el gobierno de Yucatán en 1844. Muñoz fue el encargado de propagar la vacuna en México desde 1804 y anotó las observaciones que hacía de algunos de sus pacientes. Al mismo tiempo ponía en duda las consecuencias negativas que le adjudicaban al antídoto, como enfermedades que se podían desprender de ella, o bien el hecho de que la vacuna no necesariamente protegía a la población de futuras epidemias, pues no garantizaba la inmunidad. La definió como “una enfermedad que consiste en algunos granos que les salen a estos animales en las tetas o en los pezones. Estos granos están rodeados en su base, por una inflamación rubicunda y erisipelatosa y contienen un humor claro y transparente, que no es pobre y posee la singular virtud de injertado al hombre, preservarle para siempre de la viruela maligna” (Muñoz, 1844, p.11). Asimismo, se proporcionó información sobre el desarrollo del proceso tras la inoculación en una persona, el cual transcurría aproximadamente en un lapso de diez días. Durante los primeros tres días no se observaban efectos visibles de la vacuna. En el cuarto y quinto días surgía un pequeño grano rubicundo e inflamado, que aumentaba de tamaño en el sexto día. Finalmente, entre el noveno y el décimo día, el grano alcanzaba su madurez, lo que permitía extraer de él la vacuna. El manual también detalla el procedimiento para la vacunación. Se debía seleccionar a una persona que presentara un “buen grano en su madurez”, asegurándose de que no estuviera “viciado”. Con una lanceta, se debía punzar el borde blanco del grano hasta obtener un fluido puro, apto para su aplicación, permitiendo así la inoculación de persona a persona mediante el método de brazo a brazo (Muñoz, 1844, p.11-15).

El método de vacunación de brazo a brazo se integró rápidamente a las prácticas médicas internacionales y se consolidó como un pilar fundamental de la medicina moderna en Europa y América (Agostoni, 2016, p.24). En el caso de Yucatán, la gestión del proceso de vacunación estaba a cargo de un director general, quien coordinaba las actividades con el apoyo de directores subalternos en las cabeceras de los partidos y agentes distribuidos en los pueblos bajo su jurisdicción. A cada localidad le daban una dotación de una docena de cristales destinados a la recolección del pus de los vacuníferos. Tanto los directores subalternos como los agentes recibían instrucciones precisas sobre los procedimientos a seguir. El director subalterno debía presentarse personalmente para la recepción del pus vacuno, ya que era su responsabilidad conocer el procedimiento adecuado para la apertura de los cristales y la correcta deposición del fluido en su interior. El director subalterno debía observar esta técnica y dominarla para la aplicación de la vacuna. Los directores subalternos y los agentes tenían la tarea de definir la distribución territorial de la vacunación, identificando calles, manzanas y cuarteles donde se llevaría a cabo la aplicación. Una vez administrada la vacuna, debían de registrar a las personas inoculadas, elaborando listas detalladas que incluyeran la información pertinente, dado que las personas debían ser sometidas a revacunación posteriormente. Este registro debía consignarse meticulosamente en los informes elaborados por los agentes. Los datos recopilados eran remitidos al director subalterno junto con las observaciones derivadas del proceso de vacunación, a fin de garantizar un seguimiento riguroso de la inmunización de la población (Campos, 5 ago.-22 dic. 1851, p.1-5). Aunque lo anterior corresponde a un informe elaborado en 1846, esta organización se puede también observar en las postrimerías del periodo porfiriano, cuando en cada partido del estado de Yucatán había agentes sanitarios que se encargaban de la inoculación.

Además de lo anteriormente señalado, se distribuía una cartilla de vacunación que contenía la información necesaria para su correcta aplicación. No obstante, a pesar de seguir los lineamientos establecidos en dicho documento, la vacuna no siempre resultaba efectiva en la población, como lo reportaron de manera recurrente los ayuntamientos de diversos poblados de la península (López, 12 mayo 1841, p.1-2). A esta dificultad se sumaban otros obstáculos, entre los que se destacaban la insuficiencia de recursos económicos, la resistencia de la población a recibir la vacuna, la deficiente calidad del biológico, su escasez en determinadas localidades o, en algunos casos, su pérdida total (Wan Moguel, 2023, p.87).

Estas problemáticas fueron documentadas en un informe fechado el 25 de febrero de 1848 por Antonio Rejón, director de la propagación de la vacuna en Mérida, quien señaló diversas dificultades que impedían un registro adecuado de las personas vacunadas. Destacó entre ellas el desconocimiento de los resultados de la vacunación porque las personas no acudían a una revisión que debía practicarse posterior a la aplicación del antídoto, para confirmar su efectividad y así entregarles su boleta correspondiente. También se reportaba una escasa asistencia a las sesiones de revacunación y, en términos generales, una limitada participación de la población en las campañas organizadas por el municipio (Rejón, 25 feb. 1848, p.1-2). El director de la vacunación en Mérida consideraba indispensable la implementación de medidas de “coacción” en los distintos barrios de la ciudad para fomentar la asistencia de la población a la aplicación de la vacuna, ya que estimaba que ni siquiera una sexta parte de los recién nacidos la recibían, a pesar de que debía administrarse en los primeros días de vida. Del mismo modo, propuso la difusión de los días de vacunación a través del periódico oficial, detallando el lugar, la hora y la fecha exacta de las campañas, con el propósito de garantizar un acceso más efectivo a este servicio por parte de la población (Rejón, 25 feb. 1848, p.1-2).

Es relevante destacar que, al igual que en Mérida, otras regiones de la península enfrentaron desafíos similares en la implementación de la vacunación. Un claro ejemplo de ello son las localidades de Campeche y Motul. En el caso de Campeche, las autoridades recomendaron la adopción de medidas coercitivas dirigidas a quienes se rehusaban a recibir la vacuna, atribuyendo la baja participación en las campañas al “escaso conocimiento que la población tiene de instituciones tan benéficas como la vacunación” (Campos, 13 nov.-25 dic. 1852, p.1-3).

Por su parte, en Motul, el responsable de la vacunación tenía la obligación de administrarla “invariablemente cada nueve días” para garantizar la conservación del antídoto. Sin embargo, el cumplimiento de esta tarea se veía obstaculizado por la falta de personal, ya que no contaba con agentes que lo asistieran, debiendo atender, de manera individual, a los 25 pueblos bajo su jurisdicción, los cuales se encontraban considerablemente distantes entre sí. El funcionario expresó su solicitud para la designación de agentes que apoyaran su labor; sin embargo, la escasez de recursos financieros obstaculizaba la implementación de estas medidas, ya que su salario ascendía apenas a la “mezquina” cantidad de ocho pesos mensuales (De Cepeda, 7 sep. 1852, p.1-2). Esta situación pone de manifiesto la insuficiencia de personal para llevar a cabo de manera eficiente la propagación de la vacuna, como lo pretendían las autoridades estatales, lo que generaba desafíos adicionales, entre ellos la “pérdida” del biológico. Cuando esto ocurrió, se solicitaba recurrir a otras localidades del país, e incluso al extranjero, para obtener el antídoto (Villamil, 23 feb.-24 abr. 1855, p.1-5).

Como se señaló, desde 1847 hasta 1902, la Guerra de Castas determinó que en muchas ocasiones el presupuesto se destinara a las actividades militares y se descuidaran las campañas de vacunación en los primeros años del enfrentamiento, además de que se perdiera la vacuna, como reportaron reiteradamente los galenos a cargo de esa actividad (Villamil, 23 feb.-24 abr. 1855). Es probable que esta situación esté relacionada con el devastador brote de viruela ocurrido entre 1874 y 1875, el cual provocó más de 15 mil muertes en todo el estado de Yucatán. A raíz de esta crisis, se implementaron amplias campañas de vacunación dirigidas a la población en la que debían de intervenir tanto las autoridades sanitarias como los maestros, los hacendados y los encargados de talleres artesanales. No obstante, estas medidas resultaron tardías. A pesar de los esfuerzos, los primeros intentos por establecer la obligatoriedad de la vacunación no lograron el éxito esperado. Problemas similares a los previamente mencionados continuaron manifestándose durante la segunda mitad del siglo XIX y a lo largo del siglo XX. No obstante, con el propósito de evitar que se repitieran los estragos ocasionados por la epidemia de 1874-1875, se procuró llevar a cabo campañas de vacunación de manera recurrente, como se analizará en el siguiente apartado.

En 1889 el gobierno del estado de Yucatán a cargo de Guillermo Palomino declaró obligatoria la vacuna en Yucatán y precisó que los infantes debían de recibir el antídoto dentro de sus primeros cuatro meses de edad (Wan Moguel, 2024, p.189-227). Posteriormente, en 1895 se redactó el Reglamento de la Ley de la Vacuna con diez artículos que recalcaba la importancia de propagar la vacuna por todo el estado, así como la contribución de padres, maestros y el Consejo de Salubridad en esta labor (Wan Moguel, 2024, p.396). Tiempo después, los médicos Eusebio Villamil y Manuel Pasos G. fueron comisionados por el H. Ayuntamiento de Mérida para dar cumplimiento al decreto del Gobierno del Estado, emitido en 1910 bajo la administración del gobernador Carlos Peón. En este marco se mandó publicar la *Guía práctica para el conocimiento, curación y profilaxia de la viruela*, concebida como un instrumento destinado a sistematizar saberes médicos y enfrentar los persistentes problemas sanitarios mediante la vacunación (Wan Moguel, 2024, p. 395). La aplicación de la vacuna debía llevarse a cabo tanto de forma voluntaria como coercitiva, práctica recurrente durante el porfiriato, como ha señalado Ana María Carrillo (2010). Al igual que el folleto publicado en 1843, esta guía buscaba no solo difundir el conocimiento existente, sino también ordenarlo y ponerlo al servicio de una política sanitaria más amplia orientada al control de la viruela.

El documento rescata información relevante sobre el uso del preservativo y el proceso de vacunación en niños. Se establecieron diferentes etapas en la respuesta del organismo tras la administración de la vacuna, identificándose que, a partir del quinto día, se manifestaba una vesícula o vejiguilla aplanada y transparente, la cual se expandía progresivamente hasta alcanzar su completo desarrollo en el séptimo día. A partir de este momento, el infante era denominado “vacunífero”, ya que el líquido de sus vesículas podía ser utilizado para inocular a otras personas, convirtiéndose así en un portador de la vacuna. Para ser considerado vacunífero, el niño debía contar con al menos 3 meses de edad y gozar de un buen estado de salud. Asimismo, se enfatizaba la importancia de no extraer la vacuna antes ni después del séptimo día, ni en casos donde las pústulas presentaran signos de desgarro o inflamación, con el propósito de garantizar su efectividad. Los médicos, por su parte, tenían la responsabilidad de asegurarse de que el fluido vacunal no estuviera contaminado con sangre, evitando así la transmisión de enfermedades. La normativa también incluía una serie de recomendaciones destinadas a optimizar el procedimiento y prevenir posibles complicaciones. Entre ellas, se señalaba que, en situaciones de epidemia inminente, los niños podían ser vacunados desde el nacimiento. En estos casos, si la inmunización se realizaba en los primeros días de vida, se requería una vigilancia constante en los meses posteriores para verificar su efectividad, la cual se confirmaba mediante la aparición de pústulas (Peón, 1901, p.21-29).

El manual operatorio proporcionó información detallada sobre el procedimiento de aplicación de la vacuna. En primer lugar, se utilizaba una lanceta acanalada, asegurándose de que estuviera limpia y libre de óxido. Tras secarla, se realizaba una incisión horizontal en el borde de la circunferencia del grano. Se practicaban tres punciones superficiales en la piel, de modo que únicamente se perforara la capa más externa. La lanceta debía moverse de abajo hacia arriba para que en sus bordes se acumulara la linfa vacunal. Era fundamental evitar el sangrado durante el procedimiento, pues se consideraba que la presencia de sangre incrementaba el riesgo de transmisión de enfermedades, como la sífilis. Al concluir la aplicación, la herida se cubría con parches o compresas impregnadas en solución boricada, con el propósito de prevenir infecciones (Peón, 1901, p.21-29).

## Estadísticas de vacunación

La documentación referente a la vacunación en Yucatán presenta inconsistencias, posiblemente derivadas de los problemas previamente señalados. El registro de personas inoculadas tendía a incrementarse significativamente durante los periodos epidémicos; sin embargo, una vez que la amenaza disminuía, las campañas de vacunación masiva, también se reducían. Un claro ejemplo de esto es lo ocurrido el 30 de enero de 1844, cuando se encendieron las alarmas por la presencia de un caso de viruela en el pueblo de Calotmul, en la región oriental de la península de Yucatán, lo que motivó el inicio de una campaña de vacunación tanto en esa localidad como en sus alrededores (Wan Moguel, 2023, p.91). Las primeras estadísticas sobre la vacunación en la península de Yucatán datan de los inicios del siglo XIX.^2^ En 1813, Cipriano Blanco elaboró un registro de personas vacunadas y revacunadas en Campeche. Un aspecto destacado de este documento es su clasificación de la población en calidades, como se observa en la Tabla 1.

**Tabla 1:** : Vacunados en la ciudad de Campeche, 1813

Meses	Vacunaciones	Niños españoles	Niños indios	Niños mestizos	Niños pardos	Niños negros	Totales de cada mes
Enero	**4**	39	11	43	9	–	102
Febrero	**4**	21	8	45	38	2	114
Marzo	**3**	15	11	60	5	–	91
Abril	**4**	50	9	56	3	–	118
Mayo	**4**	56	11	65	11	2	145
Junio	**4**	55	10	52	9	3	129
Julio	**4**	60	13	34	15	5	127
Agosto	**4**	12	52	40	12	6	122
Sept.	**3**	6	19	42	12	–	79
Oct.	**4**	20	16	44	20	6	106
Nov.	**4**	29	41	51	6	–	127
Dic.	**4**	33	15	39	26	3	116
Total	**46**	396	216	571	166	27	1376

Fuente: Estado..., 1814, p.1.

En las décadas posteriores, se recopilaron documentos que, en su mayoría, contienen registros nominales de los individuos vacunados. Empero, aún es necesario un análisis más detallado para evaluar el verdadero impacto de la vacunación en la población. Alcalá documentó que, en 1818, 28.773 personas fueron inmunizadas en Campeche. No obstante, se requiere un estudio más riguroso de estos datos para comprender su relevancia epidemiológica, así como relacionarlo con el número total de habitantes para entender el porcentaje total con respecto a la población, pero carecemos de esa información. En 1820, Cipriano Blanco, entonces encargado de la Junta de Sanidad, reportó 665 vacunaciones adicionales. Durante esos años, la vacuna era considerada de “buena” efectividad. No obstante, uno de los principales desafíos fue la resistencia de los padres a llevar a sus hijos a recibir la inmunización. Para garantizar el cumplimiento de las campañas de vacunación, las autoridades recomendaban amonestarlos formalmente como medida correctiva (Alcalá, 2008, p.176).

**Figura 1:**
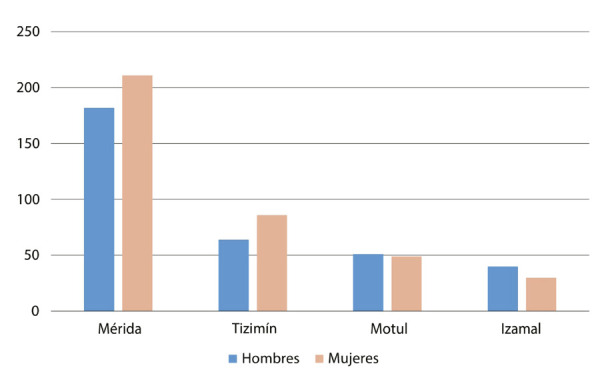
: Gráfica de los vacunados en Yucatán, 1877 (Iturralde, 1878, p.59)

Después de esas listas incompletas, los registros seriales se realizaron durante el porfiriato. Los primeros datan de 1877, cuando se anotó el número total de vacunados en algunas de las cabeceras. En ese momento había 15 partidos, solamente cuatro enviaron sus informes, pero uno llamó especialmente la atención de las autoridades; el de Ticul porque se resistió a darlos. En realidad, esa era una práctica recurrente de los que no llevaban a cabo adecuadamente la propagación de la vacuna. Por otro lado, los pocos datos levantados dejan entrever información importante, como que aún no se llevaban a cabo reportes totalmente detallados. Es decir, no hay una división por edad ni el número de vacuníferos usados, como se hará en las postrimerías del siglo XIX. Solamente se dividieron los datos por sexo: hombre y mujer y el total de los inmunizados, como se aprecia en la Figura 1.

Los registros de vacunación de 1880 fueron publicados en periódicos como *La Razón del Pueblo*, medio en el que también se anunciaban los días programados para la aplicación de la vacuna en diversas plazas y parques públicos (Canto, 1880). A partir de 1895, esta información comenzó a difundirse en *El Boletín de Higiene* y *El Boletín de Estadística*, publicaciones oficiales que desempeñaron un papel crucial en la divulgación de datos sanitarios y epidemiológicos en Yucatán. *El Boletín de Higiene* proporcionaba información detallada sobre el estado de salud y las condiciones sanitarias de la entidad, mientras que *El Boletín de Estadística* era un órgano gubernamental dedicado a la recopilación y análisis de datos relacionados con diversos aspectos, como la economía, la importación, la exportación, la mortalidad y la vacunación. Ambas publicaciones, incluían además observaciones realizadas por los responsables de la aplicación del biológico.

**Figura 2:**
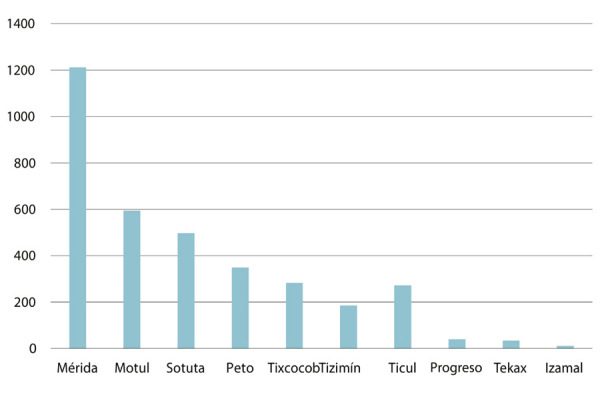
: Vacunados en el estado de Yucatán, 1895 (Castillo Peraza et al., 1896, p.167)

En los primeros meses de 1895, los informes registraron diversas problemáticas que afectaban las campañas de vacunación en distintos municipios. Entre las más significativas se encontraban la escasez o la mala calidad del preservativo, la falta de personal capacitado para su aplicación y la ausencia de niños vacuníferos. Durante el tercer trimestre de ese año, se documentó que varios municipios carecían de la vacuna, lo que impidió su aplicación. En el cuarto trimestre, se incluyó una nota en la que se señalaba: “Las dificultades inherentes a este servicio y lo difícil que ha sido conseguir tubos de linfa han hecho que la vacunación en este trimestre haya sido tan escasa. El Consejo de Salubridad se propone remediar este inconveniente” (Sales Cepeda, 1895, p.24). A pesar de esas carencias en la información recabada por las autoridades sanitarias, se puede sacar algunas conclusiones con los datos levantados de tres de los cuatro trimestres, pues en el último no se especificó la edad de los vacunados. Se observa que, de manera general, los infantes eran el tipo de población más vacunada, pues de las 2.677 personas que recibieron el antídoto, 1.807 (67,50%) eran niños de 0 a 5 años. Por otra parte, 568 (21,21%) eran menores que se encontraban en el rango de edad de los 5 a 10 años, y solamente 302 (11,28%), de 10 a 15. Llama la atención que no se haya anotado ningún adulto en las cifras oficiales. Otra de las conclusiones, es que el partido de Mérida – al igual que unos años atrás – era el lugar donde se practicaban más inoculaciones, aunque esto tiene que ver también con el número de habitantes, pues era el más poblado (Figura 2).

En 1896, persistieron las dificultades para recopilar información sobre la vacunación, lo que resultó en la recolección de datos únicamente para el primer y segundo trimestres. Mérida, capital del estado, y Peto, en el sur de Yucatán, fueron las localidades más beneficiadas con la aplicación de la vacuna (Castillo Peraza et al., 1896, p.158).

Los registros de 1897 y 1898 son fragmentarios, y en 1899 solo se documentó la vacunación correspondiente al cuarto trimestre, cuando se inmunizaron 1.244 personas en los distintos partidos de la entidad. Mérida encabezó la lista con 267 vacunados, seguida de Espita, en el oriente de Yucatán, con 238 (Castillo Peraza et al., 1900, p.11). Para los albores del siglo XX, la información se tornó más completa, en parte debido a la epidemia de 1900-1901, que provocó más de setecientos fallecimientos (Wan Moguel, 2024, p.227-234). Ante esta crisis, se emprendió una campaña de vacunación y las autoridades estatales establecieron un sistema de registro más preciso, anotando en cuadros trimestrales el número de personas inoculadas en el estado de Yucatán. Cabe señalar que, en ese momento, Campeche ya se había separado de Yucatán y Quintana Roo aún no había sido formalmente constituido como entidad federativa (Castillo Peraza et al., 1900, p.69).

**Figura 3:**
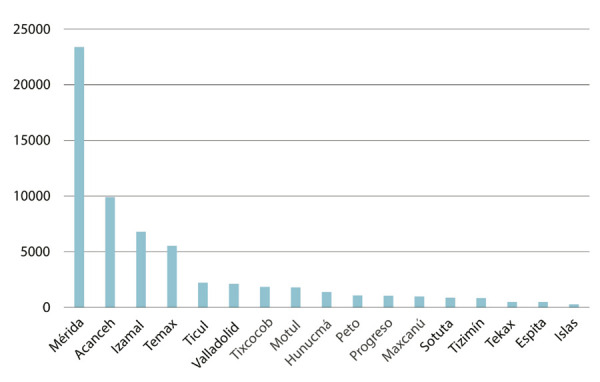
: Vacunados en Yucatán, 1900 (Castillo Peraza et al., 1900, 1901, p.24)

**Figura 4:**
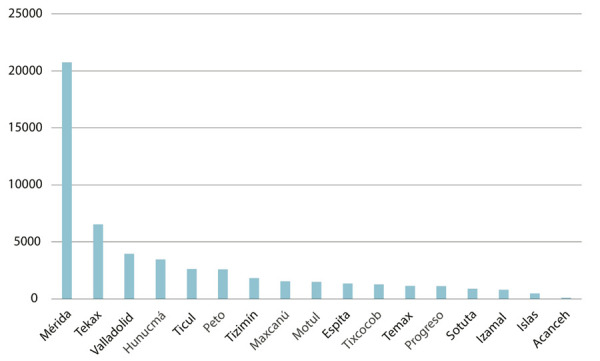
: Vacunados en el estado de Yucatán, 1901 (Castillo Peraza et al., 1901, 1902, p.13)

Como se observa en la gráfica, el partido de Mérida volvió a registrar la mayor cantidad de población inoculada, con datos correspondientes exclusivamente a los “niños de pecho” y a los mayores de un año. En el caso de los lactantes, se vacunaron 1.819 en Mérida. Según el censo de ese mismo año, había 2.648 niños en ese grupo etario (Wan Moguel, 2024, p.64), lo que indica una cobertura aproximada del 68,69%. No es posible realizar el mismo cálculo para los mayores de un año, ya que se desconoce hasta qué edad se aplicaba la vacuna, lo que podría generar inconsistencias en la estimación del porcentaje de cobertura. No obstante, es importante señalar que los datos del año siguiente también reflejan que Mérida continuó siendo el partido con mayor número de vacunaciones (Figuras 4 y 5). La situación cambió en 1902, cuando los partidos del oriente (Valladolid y Espita) y del sur (Peto y Tekax) emprendieron campañas masivas de vacunación. Es posible que este incremento estuviera relacionado con las acciones militares destinadas a sofocar la Guerra de Castas, dado que el constante movimiento de tropas pudo haber aumentado el riesgo de propagación de la viruela, incentivando así la intensificación de las campañas de inmunización.

**Figura 5:**
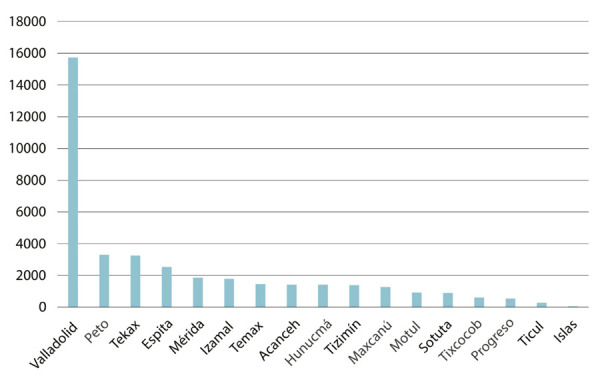
: Vacunación en el estado de Yucatán, 1902 (Castillo Peraza et al., 1902)

Aunque desde las postrimerías del siglo XIX se intentó producir la vacuna animal, no se tuvo un éxito total. En 1912, se propuso oficialmente la producción de vacuna animal como una medida para mejorar la disponibilidad y eficacia de la inmunización. Con este objetivo, el Consejo de Salubridad gestionó ante el gobernador Nicolás Cámara Vales los recursos necesarios para la creación de un instituto especializado en esta labor. Como parte de la iniciativa, se envió una delegación a Cuba para estudiar el funcionamiento del Centro General de Vacuna de La Habana. El médico designado para esta misión fue Adolfo Patrón Martínez, quien, tras su visita, formuló un proyecto para la construcción de un edificio destinado a la producción de vacuna animal en la capital del estado, Mérida. Este centro se convirtió en el primero de su tipo en todo México (Patrón Martínez, 1912).

## Consideraciones finales

La investigación realizada sobre la vacuna en Yucatán entre 1802 y 1902 refleja que su introducción y el método de “brazo a brazo” fue crucial para contener la propagación de la viruela a lo largo de un siglo. A pesar de los múltiples desafíos logísticos que enfrentaron las autoridades sanitarias, se establecieron campañas de vacunación, pero los resultados no siempre fueron fructíferos. En las primeras décadas del siglo XIX se sentaron las bases para la organización de la propagación de la vacuna. Se instalaron juntas encargadas de la labor y se enviaron a diversas regiones de la península a los vacunadores. Estas personas enfrentaron diversos desafíos como la falta de presupuesto, y la resistencia de la población a la vacunación por el miedo o la falta de información de las autoridades. Entre 1874-1875 se desencadenó una epidemia que evidencia que las campañas no necesariamente se realizaron con éxito. Sin embargo, a partir de esos años, se intensificó la propagación de la vacuna y también se publicó la Ley de Vacuna que obligaba a recibir el antídoto a los recién nacidos. En 1901, todavía se ve el interés de las autoridades por difundir los conocimientos en torno a la vacuna, que fueron plasmados en un manual donde se explicaba detalladamente el proceso de vacunación. Este estudio también vislumbró las estadísticas de vacunación que se llevaron a cabo desde los albores del siglo XIX, pero se asentaron detalladamente durante el periodo porfiriano, en el *Boletín de Estadística* y el *Boletín de Higiene*. A partir del análisis de los datos de esta fuente se vislumbró que Mérida, capital del estado de Yucatán fue el partido donde más se vacunaron a personas, pero esto cambió hacia 1900-1901, cuando los movimientos de tropas en la región sur y oriente quizá obligaron a las autoridades a difundir la vacuna para evitar posibles brotes epidémicos. Finalmente, hay que destacar que la transición hacia la producción de vacuna animal en 1912 marcó un cambio importante en el método de vacunación, aunque este tema aún se debe de analizar más detenidamente.

## Data Availability

No están en repositorio.

## NOTAS

1 No se ha encontrado información adicional de los personajes que se encargaron de la propagación de la vacuna.

2 En la actualidad el investigador Wilberth Gabriel Sánchez Moo se encuentra haciendo una amplia investigación de este periodo.
